# Is This the End of an Old Difficulty?

**Published:** 1919-04-05

**Authors:** Fred R. Spark

**Affiliations:** To Sir Wm. Middlebrook M.P., Chairman, Leeds Workpeople's Hospital Fund.


### Is this the End of an Old Difficulty?

We have been asked to print the following letter which has been written to Sir William Middlebrook, M.P.:—

LEEDS WORKPEOPLE'S HOSPITAL FUND.

*To the Editor of* The Hospital.

Dear Sir William,—You are aware that, in the year 1907, my friend, the late Archibald Ramsden, presented to me personally a cheque for £1,000, to show (as he said) his appreciation of my long public services, especially in relation to the Leeds Musical Festivals and the Leeds Workpeople's Hospital Fund. This sum was paid by me into Beckett's Bank as a separate deposit, for promoting the establishment of a convalescent home for children. Untoward events have arisen, causing long delay and preventing the fulfilment of my promise to Mr. Ramsden. I have now the pleasure of handing that gift of £1,000 to you, now the Chairman of the Fund, with the same expression as that written in a note to me with the cheque: "This sum I place in your hands, to use at your discretion, and you know that my wish is specially to assist your Convalescent Home for the Workpeople of Leeds."

I hope you will shortly be able to carry out the wishes of the donors, and thus add to the glorious achievements of the Workpeople's Fund, which I had the great pleasure of establishing in 1887. Through and by that fund there has been collected, mainly in weekly pence, over £341,000, of which the Leeds Infirmary alone has received £152,733.—Yours sincerely,

Fred R. Spark, J.P.

To Sir Wm. Middlebrook, M.P.,
Chairman, Leeds Workpeople's Hospital Fund.

[We have received an anonymous letter signed "Members of the Q.A.I.M.N.S.R." Correspondents are reminded that all letters must be accompanied by the names and addresses of the writers, not necessarily for publication, but as a guarantee of good faith.—Ed. T.H.]

